# Impact of ultrasound elastography in evaluating Bethesda category IV thyroid nodules with histopathological correlation

**DOI:** 10.3389/fendo.2024.1393982

**Published:** 2024-05-28

**Authors:** Monica Latia, Andreea Borlea, Monica Simina Mihuta, Octavian Constantin Neagoe, Dana Stoian

**Affiliations:** ^1^ Department of Doctoral Studies, Victor Babes University of Medicine and Pharmacy, Timisoara, Romania; ^2^ Dr. D Medical Center, Center for Advanced Ultrasound Evaluation, Timisoara, Romania; ^3^ Center of Molecular Research in Nephrology and Vascular Disease, Faculty of Medicine, Victor Babes University of Medicine and Pharmacy, Timisoara, Romania; ^4^ 2^nd^ Department of Internal Medicine, Victor Babes University of Medicine and Pharmacy, Timisoara, Romania; ^5^ 1^st^ Department of Surgery, Victor Babes University of Medicine and Pharmacy, Timisoara, Romania; ^6^ Second Clinic of General Surgery and Surgical Oncology, Emergency Clinical Municipal Hospital, Timisoara, Romania; ^7^ Endocrinology Unit, Pius Brinzeu Emergency Clinical Hospital, Timisoara, Romania

**Keywords:** thyroid nodule, Bethesda IV cytology, fine needle aspiration, ACR TI-RADS, thyroid ultrasound, thyroid elastography, follicular neoplasm

## Abstract

**Introduction:**

Fine needle aspiration (FNA) is the gold standard method recommended in the diagnosis of thyroid nodules. Bethesda IV cytology results are identified in 7-9% of nodules investigated through FNA, with reported malignancy rate in a wide range of 10-40%. The recommended treatment is either surgical or risk additional molecular testing before surgery. However, a large number of nodules belonging to this category (60-80%) are observed to be benign after surgical excision, which can put the patient at risk of unnecessary surgical morbidity. This study aimed to assess the diagnostic performance of conventional ultrasound, the ACR TI-RADS score and elastography in cases of Bethesda IV cytology on FNA.

**Methods:**

We evaluated ninety-seven consecutive cases with Bethesda category IV results on FNA by using conventional B-mode ultrasound, qualitative strain or shear-wave elastography (Hitachi Preirus Machine, Hitachi Inc., Japan and Aixplorer Mach 30 Supersonic Imagine, Aix-en-Provence, France) and all nodules were classified according to the ACR TI-RADS system. Conventional ultrasound was used to categorize the nodules as potentially malignant based on the following features: hypoechogenicity, inhomogeneity, a taller than wide shape, irregular margins, presence of microcalcifications, an interrupted thyroid capsule and suspicious cervical lymph nodes. Elastography classified nodules with increased stiffness as suspicious for malignancy.

**Results:**

We considered pathology results as the gold standard diagnosis, finding that 32 out of 97 nodules were carcinomas (33%) and 65 out of 97 were benign nodules (67%). The benign group included twenty cases of non-invasive follicular thyroid neoplasm with papillary-like nuclear features (NIFTP). Finally, we compared ultrasound data with pathology results, which showed that nineteen out of the 32 malignant nodules presented with increased stiffness on elastography (p=0.0002). On conventional ultrasound, we found that microcalcifications (p=0.007), hypoechogenicity and irregular margins (p=0.006) are features which can distinguish between benign and malignant nodules with statistical significance.

**Discussion:**

Integrating elastography as a parameter of the ACR TI-RADS score in the evaluation of Bethesda category IV nodules showed a sensitivity of 90.62% in detecting thyroid cancer cases (p=0.006). We can conclude that elastographic stiffness as an addition to high risk features observed on conventional ultrasound improves the detection of malignant nodules in cases with Bethesda IV cytology.

## 1 Introduction

Thyroid nodules are lesions located within the thyroid gland with an ultrasonographically distinct structure compared to the surrounding thyroid parenchyma. Thyroid nodules are a common clinical problem in the general population (1). According to epidemiological studies, the prevalence of thyroid nodules on palpation is low, of approximatively 5% in women and 1% in men living in iodine-sufficient parts of the world. However, the use of high-resolution ultrasound can detect thyroid nodules in up to 50-68% of individuals, with a higher frequency in women and the elderly (2, 3). The most important clinical issue represents differentiating benign from malignant thyroid nodules. Malignant thyroid nodules occur in 5-15% of cases, depending on age, sex, radiation exposure history, family history, and other factors (4, 5). Thyroid cancer is the most common malignant endocrine tumor and accounts for 3.4% of all cancers diagnosed annually. The vast majority of thyroid malignancies (over 90%) are due to differentiated thyroid carcinoma (DTC), which comprises papillary thyroid carcinoma (PTC) and follicular thyroid carcinoma (FTC) (6, 7).

Initial evaluation of any case where a thyroid nodule is suspected should include a physical examination of the thyroid gland and cervical lymph nodes, assessment for the presence of obstructive symptoms, family history and personal history of risk factors for thyroid cancer. Laboratory evaluation should include a serum thyroid stimulating hormone (TSH) measurement, with further tests such as free thyroxine (FT4), anti-thyroid peroxidase antibodies or TSH receptor antibodies to be used in the presence of a thyroid dysfunction (3, 8).

Ultrasonography is the preferred imaging method for assessing thyroid nodules. Diagnostic neck ultrasound, including the thyroid gland and the central and lateral compartments, should be performed in all patients suspected of having thyroid nodules, or if a nodule was incidentally detected by another imaging modality (3, 8). Ultrasound using a high-frequency, high-resolution transducer can characterize the gland and its nodules very effectively, detecting thyroid nodules of 1.0-2.0 mm, as well as stratify the risk of individual nodules which need to be further evaluated by fine needle aspiration (FNA) (3, 4). Some ultrasound parameters, such as microcalcifications, hypoechogenicity, absence of a halo, increased intranodular vascularity, nodule shape (taller than wide) or irregular margins, have been traditionally associated with an increased risk of malignancy, however, isolated ultrasound features on their own do not provide strong evidence to confirm or rule out a diagnosis of malignancy. The current guidelines recommend the use of a combination of ultrasound features to select thyroid nodules that should be biopsied (9, 10).

Risk stratification systems, established based on ultrasound characteristics, are widely applied to manage thyroid nodules and provide guidance on whether FNA should be performed (11). The American College of Radiology Thyroid Imaging Reporting and Data System (ACR TI-RADS) uses a point-based method for risk stratification and has been proven to achieve the largest decrease in unnecessary thyroid nodule FNAs. ACR TI-RADS assigns points for each of five ultrasound features (composition, echogenicity, shape, margin, echogenic foci), the sum of which determines a risk category from TR1 (benign) to TR5 (highly suspicious of malignancy) (3, 12). Recommendations for FNA or ultrasound follow-up are based on the nodule’s risk category and its maximum diameter. For risk categories TR1 through TR5 FNA should be recommended according to a size threshold. However, conventional ultrasound examination has limited value for differentiating between benign and malignant thyroid nodules due to its use of a two-dimensional technology (3, 13).

In recent years, ultrasound elastography has been developed as a new technology and applied in clinical practice with good results. Ultrasound elastography is a form of imaging used to analyze the elasticity or stiffness of tissues. The stiffness of a tissue is determined by the structural properties of its matrix. Pathological changes, such as the presence of a tumor or inflammation, alter the tissue composition and structure and increase the lesion stiffness. Thus, ultrasound elastography is a useful evaluation in distinguishing benign from malignant thyroid nodules (14). Based on the fact that on palpation a suspicious thyroid nodule is firm or hard in consistency, stiffness was adopted as indicator of malignancy for elastography (15). In clinical practice, two main thyroid elastography methods are used, depending on which physical quantities are measured: strain elastography (SE) and shear-wave elastography (SWE). SE assesses tissue elasticity through tissue displacement induced by compression. SWE assesses tissue elasticity by measuring the propagation speed of transverse shear waves (16). Considering the increased detection of thyroid nodules in the general population through the widespread use of ultrasound, elastography has emerged as an additional tool for thyroid nodule differentiation, in combination with conventional ultrasound and FNA (14, 15). Diagnostic stiffness information obtained by elastography increases the specificity and positive predictive value of conventional ultrasound (17). However, the WFUMB guideline highlights that not all malignant nodules are stiff, in particular follicular carcinomas tend to be soft or heterogeneous, therefore difficult to distinguish from benign lesions (14). Moreover, current literature provides limited data on the usefulness of ultrasound elastography in the diagnosis of Bethesda category IV nodules (18–20). Conversely, there are rich data suggesting the additional diagnostic value of elastography in thyroid nodules with indeterminate cytology, either grouping Bethesda categories III and IV together or focusing solely on Bethesda category III (21–26).

FNA remains the gold standard method used in the diagnosis of thyroid nodules, due to its high sensitivity and accuracy (27). FNA with a thin-gauge needle (25 or 27) is a safe procedure, without needing to discontinue traditional or novel oral anticoagulant therapy, as well as being cost-effective. It reduces the number of thyroid surgeries on patients with benign lesions, as well as appropriately qualifies patients with thyroid cancer for surgery (3). In 2007 the National Cancer Institute has defined the terminology and morphological criteria for the evaluation of the preparations from the FNA of the thyroid nodules, thereby creating “The Bethesda System for Reporting Thyroid Cytopathology” (TBSRTC). This system is used worldwide to classify cytology findings into six categories (Bethesda I-VI), each having a different malignant potential. Thus, it assists clinicians in understanding a nodule’s risk of malignancy to guide further management (27). The malignant potential of Bethesda category II, V, and VI nodules are well-established, each of these categories having clear recommendations for management (2). On the contrary, Bethesda category III and IV diagnoses are referred to as indeterminate, with a variable malignancy potential and their management depending on the stratification of risk factors (3). The lesions included in Bethesda category IV, defined as follicular neoplasm or suspicious for follicular neoplasm, are characterized by high cellularity, poor or absent colloid and absolute prevalence of microfollicular/trabecular structures, with characteristics suggestive of “follicular neoplasia” (3, 27). Bethesda category IV lesions account for 7-9% of nodules diagnosed through FNA and carry a malignancy rate in a wide range of 10-40% (28). The prevalence of diagnosis for Bethesda category IV has been found to differ in multiple cohorts, from as low as 1.5% to as high as 21.6% (29, 30), while a meta-analysis showed a wide range of 1.2 to 25.3% (mean 10.1%) with a risk of malignancy of 26.1% (31). The third edition of the Bethesda System was published in September 2023, with updates regarding the names of each diagnostic categories as well as their risk of malignancy (32). The cytological examination of all patients in this study was performed prior to the release of this new edition, consequently the results were classified according to the first edition of the Bethesda System (28).

Since 2016 the recommended treatment for Bethesda category IV thyroid nodules is either surgical, by total thyroidectomy or lobectomy, or risk assessment with molecular testing before surgery (2). However, a large number of these nodules (60-80%) are observed to be benign after surgical excision, meaning this group of patients is considered to be at risk of unnecessary surgical morbidity (33).

From a clinical point of view, it would be beneficial to determine which Bethesda category IV nodules are at higher risk of malignancy to better guide the management of these nodules, including the decision to undergo surgical treatment versus conservative options. Since there are studies suggestive for the added value of elastography in Bethesda category III lesions (21–25), the aim of this study is to evaluate and compare the diagnostic performance of greyscale ultrasound focusing on high risk features, the ACR TI-RADS score and ultrasound elastography in the detection of malignant nodules which were classified as Bethesda category IV after cytological testing. This will be achieved by analyzing the sensitivity, specificity, positive and negative predictive values of each method. These results might help in guiding the management of thyroid nodules with Bethesda IV cytology results in clinical practice.

The current guidelines recommend a personalized management in cases with Bethesda category IV thyroid nodules, which presents as a challenge for clinicians, who have to take into account multiple variables and decide between a large range of treatment options. The recommendation of surgical treatment is determined by the sum of identified risk factors, either through personal history, clinical examination or molecular testing. Our research raises the question: Is stiffness a viable additional risk factor in the personalized management of Bethesda category IV thyroid nodules?

## 2 Materials and methods

### 2.1 Group characteristics

This retrospective study analyzed data from patients evaluated for thyroid nodules in our Endocrinology Clinic (“Dr. D” Medical Center in Timisoara, Romania) from June 2013 until August 2023. Our research included a group of 109 patients between the ages of 25 and 84 years old, all diagnosed by FNA and with their results belonging to the Bethesda category IV. Prior to the FNA procedure, they were examined by conventional ultrasound as well as ultrasound elastography (either strain or shear-wave). Based on their cytology results all of the patients were then referred for surgical intervention, with a recommendation for total thyroidectomy. Ninety-seven of them underwent the operation while the remaining twelve either refused to do so or were lost from our evidence. The study was approved by the Ethics Committee of the Victor Babes University of Medicine and Pharmacy, Timisoara and was performed according to the ethical guidelines of the Helsinki Declaration. A written informed consent was obtained from all patients prior to inclusion.

### 2.2 Inclusion and exclusion criteria

The present study included only patients who were examined by both conventional ultrasound and elastography in our clinic and had Bethesda category IV cytology results. From the total of 590 FNAs performed in the before mentioned period, 109 cases were Bethesda category IV (18.5%). Ninety-seven of our patients were operated and provided us with the results of the histopathological examination. Out of the ninety-seven patients, sixty-five had benign pathology results and thirty-two were malignant.

**Figure 1:**
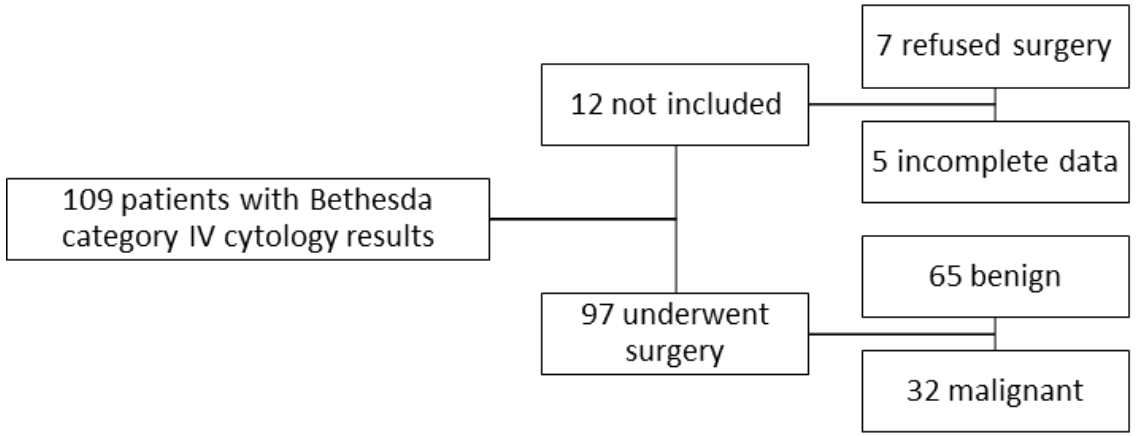
The selection process of the cases.

We excluded twelve patients who chose not to undergo surgery or who failed to provide us with the histopathology report. Patients who were radiologically examined in a different setting or did not have a complete examination by both conventional ultrasound and elastography were also excluded from this study. We did not include in this study any other Bethesda cytology findings except for category IV. The selection process of the cases is illustrated in **Figure 1**.

### 2.3 Conventional ultrasound and elastography examination

Conventional B-mode thyroid ultrasound and strain elastography were performed on the majority of the included patients (77 subjects) with a Hitachi Preirus machine with a 5-15 multi frequency linear probe (Hitachi Medical Corporation, Tokyo, Japan). All subjects were examined by the same practitioner with over 5 years of experience in thyroid ultrasound imaging. The patients were asked to lie supine and keep their neck in a slightly hyper-extended position to fully expose the anterior neck. A pillow was placed behind the neck to help with the hyperextension. Minimal pressure was applied to the neck while acquiring the images and patients were advised not to speak or swallow during the examination. The transverse and longitudinal diameters as well as thyroid volume were measured using grayscale ultrasound (34). Then the presence, location, size (three diameters and volume) and features of each thyroid nodule were assessed. To distinguish between benign and malignant sonographic features, the shape, margin, halo sign, echo structure, homogeneity, echogenicity, calcification and extra capsular invasion of each nodule were examined. The lateral compartments were screened for any abnormal lymph nodes (8). We stratified the risk of each nodule using the ACR TI-RADS system. In the same visit, real-time elastography was performed as a complementary examination to the conventional ultrasound. The patients were asked to hold their breath while the images were acquired. The probe was positioned perpendicular to the skin and light, repetitive compression was applied without lateral movement. The focal zone was placed at or below the level of the nodule. A blue-green-red color map was displayed, with red indicating soft tissue, green indicating intermediate stiffness and blue indicating high stiffness (no strain). A visual scoring system of colors was used to assess the elasticity of the nodule, in this case the 4-point elasticity score (ES) developed by Asteria et al., which showed sensitivities and specificities for thyroid cancer diagnosis of 94.1% and 81% with positive and negative predictive values of 55.2% and 98.2%, respectively (16, 35).

On a smaller proportion of the included patients (20 subjects), conventional B-mode ultrasound (following the above-mentioned technique) and shear-wave elastography were performed using the Aixplorer Mach 30 (Supersonic imagine, Aix-en-Provence, France) machine with and L 18-5 probe (linear, 5-18 MHz). We stratified the risk of each nodule using the ACR TI-RADS system. The “real-time” 2-D SWE technique was used, which allows us to place a larger ROI that can be controlled by the operator, and when activated, a color-coded map of the SWE is displayed in the field of view (FOV). One or more adjustable-measurement ROIs can be placed in the FOV. Soft tissue is displayed in blue and hard tissue is displayed in red. The patient was asked to remain still for a few seconds, a lack of motion being important for acquiring a stable elastogram. The probe was placed perpendicular to the target nodule without pressure, maintaining only slight contact with the skin and four measurements were performed. This method allows for tissue elasticity to be quantitatively estimated and is operator-independent and reproducible, with very good sensitivity and specificity for the diagnosis of thyroid cancer (14, 16, 36–38). For consistency in our study evaluating elastography in thyroid nodules, we employed a qualitative color-coded map to assess stiffness also in the SWE evaluation, similar to the approach used with strain elastography. A visual 4-point scoring system was used to assess the elasticity of each nodule (39).

The suspicious ultrasound features analyzed in this study were hypoechogenicity, inhomogeneity, a taller than wide shape, irregular margins, the presence of microcalcifications, an interrupted thyroid capsule and the presence of suspicious cervical lymph nodes. Regarding the ACR TI-RADS score, we considered categories 2 and 3 to be benign and categories 4 and 5 to be malignant. In regard to the color scoring system, we considered nodules with an ES of 1 or 2 to be benign and those with an ES of 3 or 4 to be malignant. Based on elastography findings, stiff nodules with and ES of 3 or 4 were upgraded to a higher risk category in the TI-RADS + elastography algorithm proposed in this study (18).

### 2.4 Cytological and histopathological examination

All of the cases underwent ultrasound-guided FNA, which was performed using a 25-gauge needle attached to 10 cc syringes. The patient was asked to lie on their back in a supine position with a pillow underneath their shoulders to obtain the hyperextension of the neck. The skin was thoroughly disinfected and a local anesthetic of 2 g of 5% lidocaine/prilocaine cream was applied locally. The transducer had a sterile cover for the protection of the patient. Firstly, the nodule was located by ultrasound, then the needle was inserted parallel to the probe, using the anterior technique. Confirmation of the needle entering the nodule was observed on ultrasound. The cellular material was gathered using “coring” movements of the needle within the nodule. Each nodule was punctured at least twice, and the obtained material spread on slides (minimum 5 slides per nodule). The slides prepared by aspiration were fixed using 95% ethyl alcohol and stained using May-Grunwald-Giemsa stains for cytological evaluation. All patients signed an informed consent for FNA biopsy. All of the slides containing FNA results were analyzed by the same cytologist with an expertise in thyroid pathology and the Bethesda System for Reporting Thyroid Cytopathology was used to report the results. In this study we included only findings pertaining to the Bethesda category IV.

Total thyroidectomy was recommended to all patients, and was performed by experienced surgeons. All of the surgically resected specimens underwent histopathological examination according to standard procedures. Thyroid tissues were manually processed and fixed in 10% neutralized formaldehyde. Nodules suspected for malignity were embedded in paraffin and sectioned to obtain a thickness of 4 µm. Each tissue section was stained using hematoxylin and eosin (H&E). A team of experienced pathologists from the Pathology Department performed the histological analysis of the slides.

### 2.5 Data analysis

Microsoft Excel was used for data collection and MedCalc Statistical Software version 20.111 (MedCalc Soft-ware Ltd., Ostend, Belgium) was used for the statistics analysis. The analysis focused on the performance of greyscale ultrasound parameters, the ACR TI-RADS algorithm, elastography and the association of ACR TI-RADS and elastography in distinguishing between benign and malignant thyroid nodules. We determined the true-positive (TP), true-negative (TN), false-negative (FN) and false-positive (FP) diagnoses. Sensitivity (Se) was calculated as TP/(TN+FP), specificity (Sp) as TN/(TN+FP), accuracy as TP+TN/total cases, positive predictive value (PPV) as TP/(TP+FP) and negative predictive value (NPV) was calculated as TN/(TN+FN). Fisher exact 2-tailed test and multiple regression analysis were used for statistical analyses. Area under the receiver operated characteristic curve (AUC-ROC) analysis was performed to evaluate the sensibility and specificity of each parameter in relation to the presence of cancerous nodules. The Fisher’s exact test was used to determine whether any of the analyzed ultrasound and elastography parameters are more likely to be present in malignant versus benign lesions. Prior to the analysis of numerical data, the normality of data distribution was tested using the Shapiro-Wilk test. Consequentially, we performed statistical tests appropriate for the normality of the data in question: medians and the Mann-Whitney test were used to describe the non-normally distributed data. The Pearson correlation coefficient (Phi coefficient) was employed to evaluate the strength and direction of association between binary parameters. The significance threshold was considered a p-value of less than 0.05.

## 3 Results

The 97 participants were divided into two groups according to the histological examination of their thyroid nodules, malignant *vs* benign (see **Table 1**). Out of the 65 patients (67%) in the benign group, 20 (20.6%) had a histopathological result of non-invasive follicular thyroid neoplasm with papillary-like nuclear features (NIFTP). The cancer group included 32 patients (33%), 21 of them with follicular thyroid carcinoma (FTC) (21.6%), 8 with papillary thyroid carcinoma (PTC) (8.2%) and 3 cases of papillary thyroid microcarcinoma (microPTC) (3.1%); for the further statistical analyses we included PTC and microPTC cases in the same group of 11 patients (11.3%).

**Table 1:** Distribution of cases according to histopathology results.

Pathology diagnosis	No.	%
Benign	65	67%
*NIFTP*	20	20.6%
Malignant	32	33%
*FTC*	21	21.6%
*PTC*	8	8.2%
*MicroPTC*	3	3.1%

No., number of cases; %, percentage of cases; NIFTP, non-invasive follicular thyroid neoplasm with papillary-like nuclear features; FTC, follicular thyroid carcinoma; PTC, papillary thyroid carcinoma; MicroPTC, papillary thyroid microcarcinoma.

Out of our total 97 patients, 82 were females (84.5%) and 15 were males (15.5%). The cancer group included 23 females (23.7%) and 9 males (9.3%), while in the cancer-free group only 6 of the patients were males (6.2%) and 59 were female (60.8%), as shown in **Table 2**. The AUC-ROC analysis showed that gender has significant specificity in discriminating between cancerous and benign thyroid nodules, but low sensitivity (AUC=0.59, p=0.03, Se%=28.12, 95% CI=13.7 - 46.7, Sp%=90.77, 95% CI=81.0 - 96.5, PPV%=60, NPV%=72). With regard to age, the median age of the 97 participants was 48 years (limits 25-84 years, see **Table 2**). No significant differences were detected between the two groups, the median age in the cancer group was 49 years (limits 25-82 years), while in the cancer-free group it was 47 years (limits 27-84 years), p=0.75. The AUC-ROC analysis for age showed that it is not a reliable distinguisher between benign and malignant nodules, p=0.76, with a satisfying sensitivity, but an extremely low specificity (cut-off ≤66 years, AUC=0.52, Se%=78.1, 95% CI=60.0 - 90.7, Sp%=9.23, 95% CI=3.5 - 19.0, PPV%=29.8, NPV%=46.2).

**Table 2:** Distribution of cases according to age and gender.

Characteristic	All (n=97)	Benign (n=65)	Malignant (n=32)
Median age, y	48 (25-84)	47 (27-84)	49 (25-82)
Gender, n (%)			
Female	82 (84.5%)	59 (60.8%)	23 (23.7%)
Male	15 (15.5%)	6 (6.2%)	9 (9.3%)

n, number of cases; %, percentage of cases; y, years.

With regard to the nodular maximal diameter and nodular volume as possible discriminators between benign and malignant nodules, the AUC-ROC analysis revealed good sensitivities, but low specificities, and the p-values did not reach significance. Malignant nodules presented a higher median for maximal diameter than benign ones (2.25 cm *vs* 1.9 cm), but the significance threshold was not met (p=0.78). Moreover, cancerous nodules also presented a higher volume than benign ones, with median values of 3.38 ml *vs* 2.01 ml, but the difference was still not statistically significant (p=0.46), as illustrated in **Table 3**.

**Table 3:** The area under the receiver operating characteristic curve analysis and cut-off values for nodular maximal diameter and nodular volume in distinguishing between benign and malignant Bethesda category IV nodules.

	Criterion (cut-off)	AUC	*p*	Sensitivity %	95% CI	Specificity %	95% CI	PPV %	NPV %
Maximal diameter (cm)	>1.74	0.51	0.78	71.87	53.3 - 86.3	47.69	35.1 - 60.5	40.4	77.5
Volume (ml)	>1.46	0.54	0.45	71.87	53.3 - 86.3	47.69	35.1 - 60.5	40.4	77.5

AUC, area under the curve; CI, confidence interval; PPV, positive predictive value; NPV, negative predictive value; cm, centimeters; ml, millilitres.

While all ultrasound features considered as suspicious for malignancy, as well as elastographic stiffness (ES scores 3 and 4) were overall more prevalent in cancerous lesions, the statistical analysis revealed significant results only for microcalcifications (p=0.001) and elastographic stiffness (p=0.0004). Consequently, the presence of microcalcifications and lesions presenting as stiff on elastography could be used as indicators of malignancy in the evaluation of Bethesda category IV thyroid nodules. **Table 4** presents descriptive data regarding the presence of each ultrasound feature suspicious for malignancy and elastographic stiffness in the two analyzed groups, as well as the results of the Fisher’s exact test used to compare them.

**Table 4:** The analysis of ultrasound characteristics for each nodule examined; the Fisher’s exact test determines whether the analyzed US characteristics are more likely to be present in one of the two groups.

Nodular US characteristic	Malignant total n=32 cases		Benign total n=65 cases		Fisher’s exact test *p*
	%	n	%	n	
Taller than wide	21.9	7	15.4	10	0.39
Hypoechogenicity	90.6	29	69.2	45	0.27
Inhomogeneity	62.5	20	56.9	37	0.71
Irregular margins	34.4	11	9.2	6	0.63
Interrupted capsule	9.4	3	1.5	1	0.99
Suspicious LN	12.5	4	4.6	3	0.99
Microcalcifications	21.9	7	1.5	1	**0.001**
Stiffness	59.4	19	21.5	14	**0.0004**

US, ultrasound; n, number of cases; %, percentage of cases; LN, lymph nodes.

The significant results in this table were bolded.

**Figure 2:**
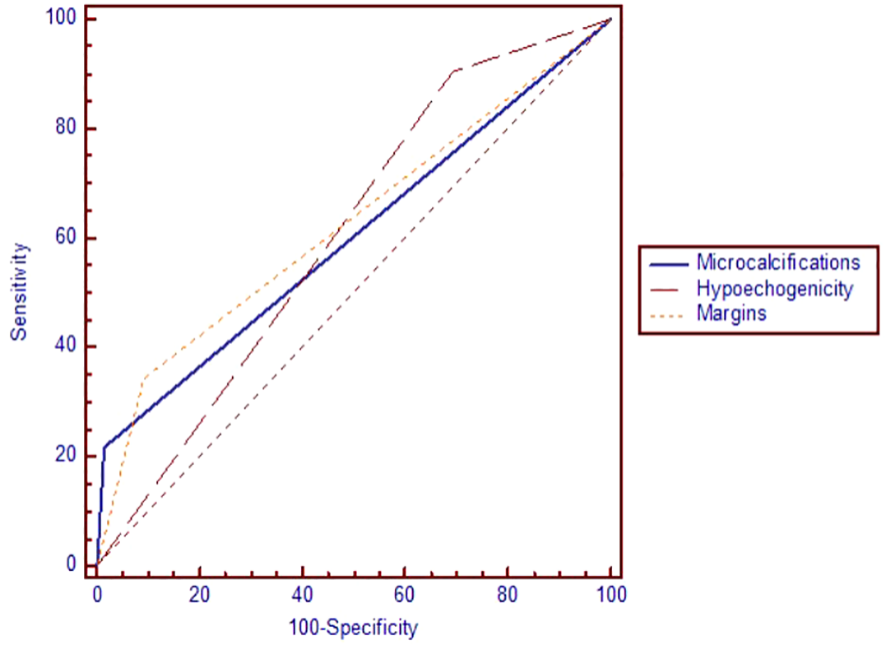
The area under the receiver operating characteristic curve for microcalcifications, hypoechogenicity and irregular margins.

In terms of the performance of greyscale ultrasound in distinguishing between benign and malignant nodules, we observed that three of the seven high-risk features were statistically significant according to the AUC-ROC analysis: the presence of microcalcifications (p=0.007), hypoechogenicity and irregular margins (p=0.006), as seen in **Figure 2** and **Table 5**. Microcalcifications were highly specific for cancer (Sp%=98.5) and were present in 7 of our malignant nodules. Hypoechogenicity, although with a relatively low specificity, has a significantly high sensitivity and was present in all cases of PTC (Se%=100) and in 18 out of 21 cases of FTC (Se%=85.7). In contrast, irregular or lobulated margins which were present in 11 of the malignant nodules, were observed to have a significant specificity but a quite low sensitivity. In terms of the other four features for which we didn’t find statistically significant data, nodules with a taller than wide shape comprised 7 out of all cancers, this feature presenting a relatively high specificity but a quite low sensitivity. Inhomogeneity was found to offer quite modest results in terms of both sensitivity and specificity; it characterized 20 out of our 32 malignant nodules. The presence of an interrupted capsule and of suspicious lymph nodes were both highly specific features (Sp%=94.46 and Sp%=95.36, respectively), but because of their low prevalence, had a low sensitivity.

**Table 5:** The area under the receiver operating characteristic curve analysis for the performance of ultrasound parameters in distinguishing between benign and malignant thyroid nodules.

US Parameter	AUC	*p*	Sensitivity %	95% CI	Specificity %	95% CI	PPV %	NPV %
Taller than wide	0.532	0.064	21.9	9.3 - 40.0	84.62	73.5 - 92.4	41.2	68.8
Hypochogenicity	**0.606**	**0.006**	**90.62**	**75.0 - 98.0**	**30.77**	**19.9 - 43.4**	**39.2**	**87**
Inhomogeneity	0.527	0.6	62.50	43.7 - 78.9	43.08	30.8 - 56.0	35.1	70
Irregular margins	**0.625**	**0.006**	**34.38**	**18.6 - 53.2**	**90.77**	**81.0 - 96.5**	**64.7**	**73.7**
Interrupted capsule	0.549	0.15	9.38	2.0 - 25.0	98.46	91.7 - 100.0	75	68
Suspicious LN	0.539	0.22	12.50	3.5 - 29.0	95.38	87.1 - 99.0	57.1	68.9
Microcalcifications	**0.601**	**0.007**	**21.9**	**9.3.0 - 40**	**98.5**	**91.7 – 100**	**87.5**	**71.9**
Stiffness	**0.689**	**0.0002**	**59.4**	**40.6 – 76.3**	**78.5**	**66.5 – 87.7**	**57.6**	**79.7**
ACR TI-RADS	0.575	0.082	84.4	67.2 - 94.7	30.8	19.9 – 43.4	37.5	80
ACR TIRADS + ES	**0.606**	**0.006**	**90.62**	**75.0 - 98.0**	**30.8**	**19.9 – 43.4**	**39.2**	**87**

US, ultrasound; AUC, area under the curve; CI, confidence interval; PPV, positive predictive value; NPV, negative predictive value; ACR TI-RADS, American College of Radiology Thyroid Imaging Reporting and Data System; LN, lymph nodes; ES, elastography.

The significant results in this table were bolded.

The Pearson (Phi) coefficient was calculated between ultrasound parameters and elastography. A moderate, positive association was found between elastography and echogenicity, of approximately 0.408 (p<0.0001). Another positive, weak to moderate correlation was established between elastography and ACR-TIRADS category (phi=0.387, p<0.001), and a positive, weak correlation with homogeneity (phi=0.260, p=0.01).

The statistical significance threshold was not met for the ACR TI-RADS score to be used as standalone risk assessment method (see **Table 5**). With regard to elastographic stiffness, we found a satisfying specificity, but quite average sensibility (AUC=0.68, p=0.0002, Se=59.4%, Sp=78.5, PPV=57.6%, NPV=79.7%). Considering elastography color scores 1 and 2 as suggestive for benignity and scores 3 and 4 as predictors for malignancy, stiffness evaluation helped us identify 64 nodules belonging to the benign group (score 1 and 2) and 33 nodules to the malignant group (scores 3 and 4). Less than half of the cases (13 out of 32) with a histological diagnosis of malignancy were characterized as soft based on elastography (scores 1 and 2). Overall, the prevalence of cancer in the soft lesions (scores 1 and 2) was 20%. As for stiff lesions, 57% of scores 3 and 4 cases proved to be malignant.

**Figure 3:**
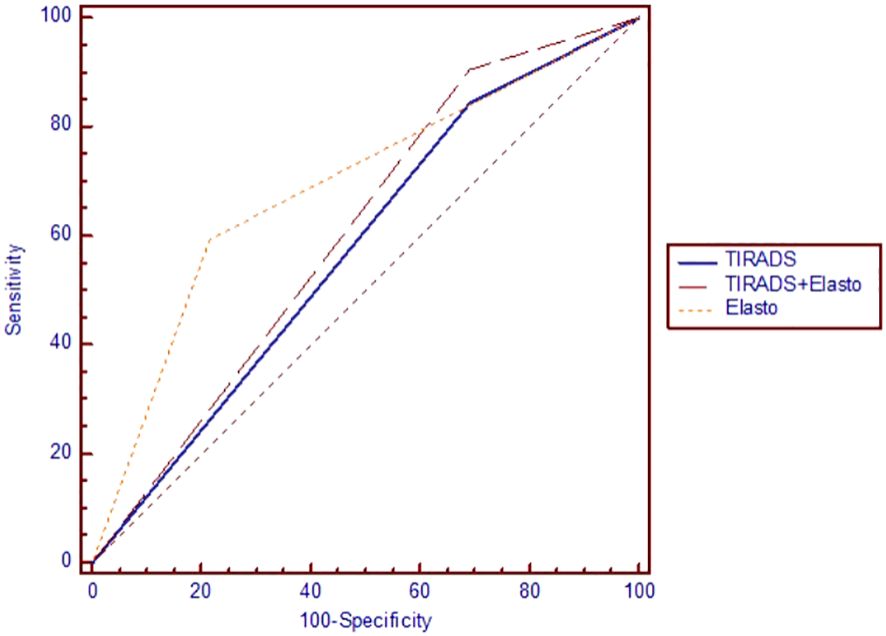
The area under the receiver operating characteristic curve for ACR TI-RADS and elastography.

**Figure 4:**
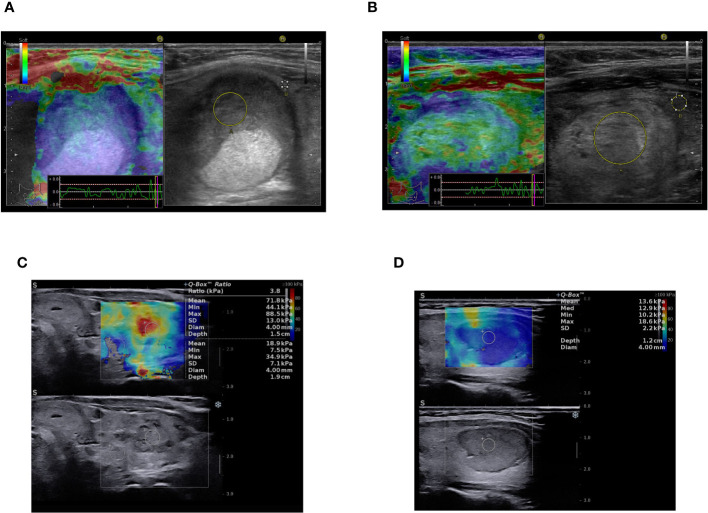
Multiparametric ultrasound-based evaluation: **(A)** B-mode image on the right - solid, oval, inhomogeneous, hypoechoic thyroid nodule with smooth margins, ACR-TIRADS 4, strain elastography color-map on the left - stiff in elastography (color code blue), asteria 4, ACR-TIRADS + elasography score 5, Histopathology: NIFTP. **(B)** B-mode image on the right - solid, oval, inhomogeneous, isoechoic thyroid nodule with smooth margins, ACR-TIRADS 3, strain elastography color-map on the left - mostly soft in elastography (code green), asteria 2, ACR-TIRADS + elasography score 3, Histopathology: NIFTP. **(C)** B-mode image on the bottom - solid, oval, inhomogeneous, hypoechoic thyroid nodule with ill-defined margins, ACR-TIRADS 4, SWE elastography color-map on the upper side of the image - stiff in elastography (code red), asteria 4, ACR-TIRADS + elasography score 5, Histopathology: Follicular thyroid carcinoma. **(D)** B-mode image on the bottom - solid, oval, homogeneous, hypoechoic thyroid nodule with smooth, lobulated margins, ACR-TIRADS 5, SWE elastography color-map on the upper side of the image - mostly soft in elastography (code blue-yellow), asteria 2, ACR TIRADS + elasography remains score 5, Histopathology: Follicular adenoma.

Testing the ROC curves regarding differences between the ACR-TIRADS score, elastographic stiffness and ACR-TIRADS + elastography, rendered the results depicted in **Figure 3**. Comparing ACR-TIRADS *vs* ACR-TIRADS + elastography curves did not reveal significant differences, p=0.15. Neither did comparing ACR-TIRADS *vs* elastographic stiffness (p=0.77), nor ACR-TIRADS + elastography *vs* elastographic stiffness (p=0.62). However, the AUC-ROC analysis shows that the combined use of the ACR TI-RADS score (which is a sum of high risk features observed on conventional ultrasound) with elastographic stiffness as a complementary method for risk upgrade represents the most sensitive diagnostic algorithm for detecting cancer (AUC=606, p=0.006) (see **Table 5**). **Figure 4** illustrates the simultaneous evaluation of the thyroid nodules included in this study through B-mode ultrasound and elastography (strain and shear-wave).

## 4 Discussion

Ultrasound is the preferred method for the detection of thyroid nodules and in necessary cases can be followed up by ultrasound-guided FNA to gain information for further medical management. FNA helps to reduce unnecessary surgeries and identifies nodules with a high risk of malignancy. The cytology findings of FNA are classified according to the Bethesda system. In case of nondiagnostic or unsatisfactory thyroid FNA results, ultrasound findings can guide the pathologist to manage these particular thyroid nodules (40). However, the risk of malignancy for thyroid nodules classified as Bethesda category IV varies widely. Our study explored whether certain characteristics of thyroid nodules on both ultrasound and elastography can facilitate clinicians to interpret the risk for malignancy of Bethesda category IV nodules and aid in their further management.

Malignancy rates of Bethesda category IV nodules vary from institute to institute, higher rates more commonly found in multicentric studies, from as low as 16.8% (41) to as high as 72.4% (42), however multiple studies reported malignancy rates between 25-50% (43–45). The present study evaluated a cohort of 97 patients with Bethesda category IV cytology findings and a complete histological report. Our results found the malignancy rate for Bethesda category IV nodules (33%) to be only slightly higher than what was outlined by the 2017 Bethesda System for Reporting Thyroid Cytopathology guideline (28) and similar to more recent studies with a large cohort (46), indicating that our nodules were correctly characterized based on cytology, avoiding selection biases. In our series we found 20.6% of NIFTP cases after histological examination, cases which are usually clustered within Bethesda categories III, IV and V, rendering our findings consistent with previous studies (47, 48). However, among the cancer group, follicular cancer was the most common malignant variant, followed by papillary thyroid cancer, which is in contrast to the histopathological distribution reported by other investigators and could be explained by a lower number of cases (43, 49). Malignancy rates carry an important weight in guiding clinicians and surgeons to select cases for surgical treatment or observation and follow-up (50).

In the present study, the median age was 48 years and no significant differences between the cancer and cancer-free group were observed, with a greater proportion of females (82%) compared to males, which is comparable to other published studies (44, 50). According to our results, age cannot be used as a reliable factor in predicting malignancy, findings which differ from several others in specialized literature, where younger age was identified as an independent risk factor for thyroid cancer in indeterminate nodules and an inverse correlation between patient age and malignancy risk for nodules undergoing FNA was reported (51–53). However, we did find that female gender is a significant risk factor for malignancy, which is in line with other studies (53, 54). In terms of thyroid nodule size, our medians of 2.25 cm for malignant nodules and 1.9 cm for benign ones are comparable to other reports (54, 55), but a significant association between nodule size and risk of malignancy cannot be identified, a finding consistent with the results of other investigations (52, 55).

In general, ultrasound features suspicious for malignancy in indeterminate thyroid nodules include hypoechogenicity, inhomogeneity, irregular margins, a taller than wide shape, solid composition, the presence of microcalcifications and extra-capsular extension (2, 56, 57). It was reported that irregular margins, hypoechogenicity and a taller than wide shape were all associated with malignancy in thyroid nodules, the highest positive predictive value being attributed to irregular margins (56, 58). However, specialized literature reports a variable utility of these ultrasound features, with some studies suggesting their potential utility and others stating that specific ultrasound features are poorly predictive of malignancy when used singularly (59, 60). Our results confirm the findings of several studies, which showed hypoechogenicity to be the only feature with a sensitivity of 87.2%, comparable to our finding of 90.62% (61, 62). Multiple studies also report that the presence of microcalcifications and irregular margins are both features that show a high specificity for malignancy, ranging from 90.8% to 96.1%, which was also observed in our study (61, 63), microcalcifications being the feature with the highest specificity (98.5%). It has been demonstrated that a taller than wide shape is the most accurate ultrasound feature for judging the malignancy of a thyroid nodule (57, 60, 62). In our study, we also observed a relationship between the taller than wide nodular shape and the risk of malignancy. Extra-capsular extension is highly reliable sign of malignancy as well as an unfavorable prognostic sign (64). Minimal extra-capsular extension can be suspected on ultrasound examination in the presence of border abutment, contour bulging, or loss of the echogenic border, however clinicians need to be cautious when reporting minimal extension, particularly in case of otherwise benign-appearing nodules (65, 66). Ultrasound examination includes cervical lymph node evaluation, which should be described as normal, indeterminate, or suspicious, and located using the six cervical levels nomenclature. Lymph nodes of high suspicion present cystic areas, microcalcifications, thyroid tissue-like appearance, and anarchic vascularity with the absence of a visible hilum (8, 67). According to multiple studies, the co-presentation of hypoechogenicity, irregular margins, and microcalcifications was associated with a greater risk of cancer than any of these features used in isolation, and had a higher specificity but lower sensitivity for diagnosing malignancy (44, 52, 57, 60).

With regard to ultrasound risk stratification systems, which offer guidance in assessing thyroid nodules and selecting those which need to be further evaluated by FNA (68, 69), we have chosen for our research the point-based ACR TI-RADS scoring system, as it was demonstrated to have the highest diagnostic performance, significantly superior to American Thyroid Association guidelines (70). The question was raised for the possible use of the EU-TIRADS system, taking into account the geographic area of our patients, but further research showed that the ACR TI-RADS system provides a better performance (71). Our research revealed a lower sensitivity, specificity and PPV for the ACR TI-RADS system compared to the results observed in similar studies, due to a high proportion of the nodules in our benign group being described as solid and hypoechogenic and thus classified as ACR TI-RADS category 4, which we considered as suggestive for malignancy. However, our findings are in agreement with the previously mentioned studies, that the ACR TI-RADS system is not able to yield a statistically significant correlation between ultrasound risk category and malignancy (72, 73). Recent data underlines that combining the ACR-TIRADS algorithm with either strain or shear-wave examination maximizes the correct classification of thyroid nodules with similar accuracy between them, while the type of elastography can be chosen according to the preferences of each clinician (74).

The diagnostic performance of evaluating thyroid nodules belonging to intermediate categories, including Bethesda category IV, by ultrasound only has shown poorer outcomes, compared to the nodules classified in the other Bethesda categories, thus highlighting the importance of adding other evaluation methods to these cases (75). Data recorded in multiple studies shows that the majority of Bethesda category IV lesions present as heterogeneous on ultrasound, with usually unsuspicious features, meaning that standalone ultrasound characteristics and the ACR-TIRADS algorithm, while reliable methods to detect papillary thyroid carcinoma, have a lower accuracy for other histological types, including follicular lesions. Due to the reported lack of suspicious ultrasound features in follicular thyroid carcinoma, and acknowledging the limitations of the cytological evaluation in its detection, it is prudent to exercise caution when employing ultrasound for the management of nodules classified as Bethesda category IV (76, 77).

Risk predictions were enhanced by the combined use of conventional ultrasound characteristics with the elastography color score (18–22). Elastography, used as a parameter of the TI-RADS score, increases the diagnostic accuracy of patients with indeterminate thyroid cytology and may lead to a reduction of surgeries in cases with low stiffness (23). In the present study, by including elastography as a parameter of ACR TI-RADS through upgrading nodules described as stiff on elastography (scores 3 and 4), we noticed an increase in specificity (from 84.37% to 90.62%) as well as in the negative predictive value (from 79.2% to 84.2%), which is in line with other findings (23, 24). As mentioned above, since a significant proportion of our ACR-TIRADS score 4 nodules were benign, therefore lowering the specificity of the algorithm itself, only a modest improvement in specificity could be observed when adding stiffness as an additional risk factor. These results reconfirm that including elastography as a parameter of the ACR TI-RADS score can help with the selection of malignant nodules in case of Bethesda IV cytology, thus avoiding unnecessary surgery in these cases, as well as optimizing their personalized management, which can present as a challenge for the clinician (25, 26).

## 5 Conclusions

Our results demonstrate that while certain ultrasonographic characteristics—like microcalcifications, hypoechogenicity, and irregular margins, along with elastographic stiffness—provide valuable insights into detecting malignancy among Bethesda IV cytology nodules, integrating elastography into the ACR TI-RADS scoring system emerges as the most sensitive diagnostic approach. This method effectively identifies malignant nodules, thereby reducing the incidence of unnecessary surgeries. Considering stiffness as a risk factor further refines ultrasound-based diagnostic accuracy even in this particular cytological category.

## Data availability statement

The raw data supporting the conclusions of this article will be made available by the authors, without undue reservation.

## Ethics statement

The studies involving humans were approved by The Ethics Committee of the Victor Babes University of Medicine and Pharmacy. The studies were conducted in accordance with the local legislation and institutional requirements. The participants provided their written informed consent to participate in this study.

## Author contributions

ML: Conceptualization, Data curation, Formal analysis, Investigation, Methodology, Writing – original draft. AB: Conceptualization, Methodology, Project administration, Validation, Visualization, Writing – review & editing. MM: Formal analysis, Software, Validation, Visualization, Writing – review & editing, Data curation. ON: Project administration, Resources, Writing – review & editing, Supervision. DS: Conceptualization, Methodology, Resources, Supervision, Validation, Writing – review & editing.
